# Correlation between periodontitis and prostate-specific antigen levels in the elderly Chinese male population

**DOI:** 10.1186/s12903-022-02171-9

**Published:** 2022-05-06

**Authors:** Mengyun Mao, Haihua Zhu, Yanyi Xie, Da Ni, Fudong Zhu, Qianming Chen

**Affiliations:** Stomatology Hospital, School of Stomatology, Zhejiang University School of Medicine, Zhejiang Provincial Clinical Research Center for Oral Diseases, Key Laboratory of Oral Biomedical Research of Zhejiang Province, Cancer Center of Zhejiang University, Hangzhou, 310006 China

**Keywords:** Periodontal disease, Periodontitis, Prevention, Risk, PSA

## Abstract

**Background:**

The comparison of prostate-specific antigen (PSA) levels among older individuals with different periodontal statuses has not been fully investigated. Here we aimed to explore the correlation between the staging and grading of periodontitis and PSA levels in an elderly Chinese male population, which may lead to a biopsy recommendation and prevent prostate cancer as early as possible.

**Methods:**

The study included 996 individuals aged ≥ 55 years who participated in routine postretirement physical examinations. Periodontal data included probing depth and gingival recession on four sites/tooth and on two diagonal quadrants (1–3 or 2–4) while excluding third molars, and clinical attachment loss was calculated. Periodontal status was classified as none, mild-moderate or severe periodontitis according to the Centers for Disease Control and Prevention and the American Academy of Periodontology case definition. Blood samples, oral health status and sociodemographic characteristics were collected by using general and oral examinations and questionnaires. Linear and logistic regressions were used to estimate the correlation between periodontitis severity and PSA levels, respectively.

**Results:**

A total of 479 men had mild-moderate periodontitis and 355 had severe periodontitis; 162 men were periodontally healthy individuals. After adjusting for potential confounders, PSA levels were significantly lower in the individuals without periodontitis than in those with mild-moderate (*P* = 0.04) or severe (*P* = 0.03) periodontitis. However, PSA levels did not significantly correlate with periodontitis severity (*P* = 0.06). Although the ORs of elevated PSA were not significant, individuals with PSA ≥ 4.0 ng/mL were more likely to have periodontitis.

**Conclusions:**

In a sample of an elderly Chinese male population, after adjusting for possible confounders, serum PSA levels in individuals with periodontitis were significantly higher than those in individuals without periodontitis, but serum PSA did not significantly correlate with periodontitis severity.

## Background

Periodontitis is one of the most frequent diseases with a prevalence of 45–50% of the world population [1], in which inflammation occurs in the periodontal tissue. In mainland China, the incidence of periodontitis in people over 55 years old is 64.6%, and the incidence of severe periodontitis is 37.3% [2]. On the tooth surfaces, specific pathogens contribute to the formation of complex communities organized as biofilms by binding to early colonizing, which initiates gingival inflammation. Subsequently, oral bacterial dysbiosis results from gingival inflammation and bacteria by producting enriched species. Afterward, host inflammatory and immune mechanisms occur in response to uncontrolled bacterial challenge. The relevant tissues are ultimately changed in the case of gingivitis and periodontitis resulting from host–microbial interactions [3]. As the inflammation process continues, the tissues and bones that support the teeth are eventually destroyed [4]. Periodontitis can clinically manifest as gingival bleeding, periodontal pocket formation, and tooth loosening and loss, which seriously affect the quality of life of patients [5, 6]. It has been widely investigated by researchers that periodontal pathogens are associated with various nonmalignant and malignant diseases, such as cardiovascular diseases, Alzheimer's disease, diabetes, lung cancer and other malignant diseases [3, 7, 8].

Prostate cancer (PC) is an epithelial malignant tumor of the prostate gland. The incidence rates of PC vary geographically and are lower in East Asia [9]. However, the incidence rates have increased significantly in China due to economic growth, prolongation of life span and culture exchange [10, 11]. In 1980, Papsidero et al*.* was the first group to quantify prostate-specific antigen (PSA) levels in the blood of humans [12], constituting the beginning of the clinical application of PSA as a marker, which is widely used to screen PC in medicine today. It has been established that the PSA cutoff recommended for biopsy is 4.0 ng/mL, although many cases have been missed with this cutoff [13]. Scholars in the United States suggested a limit of 2.5 ng/mL to increase sensitivity [14].

Recently, Joshi et al. found a relationship between periodontitis and PSA levels in chronic prostatitis patients [15]. Boyapati et al*.* showed a pathological link between moderate-to-severe prostatitis and periodontitis [16]*.* Estemalik et al. also reported the presence of *Porphyromonas gingivalis* and *Treponema denticola* DNA in the expressed prostatic secretion and dental plaque of the same patient, supporting the relationship between periodontitis and prostatitis [17]. In addition, scholars [18] have revealed that PSA levels could be reduced after the treatment of patients with chronic periodontal inflammation. However, Kruck et al*.* suggested no influence of chronic periodontitis treatment on tPSA or fPSA levels in asymptomatic men [19]. The comparison of PSA levels with different periodontal statuses has not been fully investigated, especially in the elderly population. The severity of periodontitis positively correlates with age [2]. The European Randomized Study of Screening for Prostate Cancer (ERSPC) and Prostate, Lung, Colorectal and Ovarian (PLCO) cancer screening trials have indicated that PSA levels in men 55 to 74 years old are ≥ 3.0 ng/mL in approximately 14–16% [20]. "Early screening, early diagnosis and early treatment" is an effective strategy to reduce and prevent the mortality of cancer patients [21]. At present, PSA levels are an important indicator of PC biopsy screening, and the correlation with periodontitis in the elderly Chinese male population of the PC screening age range is not clear. Since the oral cavity is easily accessible, it is used for the early diagnosis of many nonoral diseases. Therefore, we aimed to investigate the correlation between the staging and grading of periodontitis and PSA levels in an elderly Chinese male population without PC, which may lead to a biopsy recommendation in an effort to prevent PC as early as possible.

## Materials

### Study design

People who have retired from urban community enterprises in China have the opportunity to undergo regular physical examinations in community hospitals by appointment, which includes blood tests and systemic disease examinations. The stratified multistage cluster random sampling method according to the district, street, and community was used in our survey. Six community hospitals in Hangzhou were randomly chosen, and 5819 55–90-year-old residents who underwent routine physical exams from November 2021 to December 2021 in these hospitals were used as the source population. Participants aged ≥ 55 years were eligible for oral examination if they did not refuse to participate or meet any health exclusion criteria. Individuals who had fewer than 6 teeth or conditions in which they could not tolerate oral examinations were excluded.

### Population

**Fig. 1:**
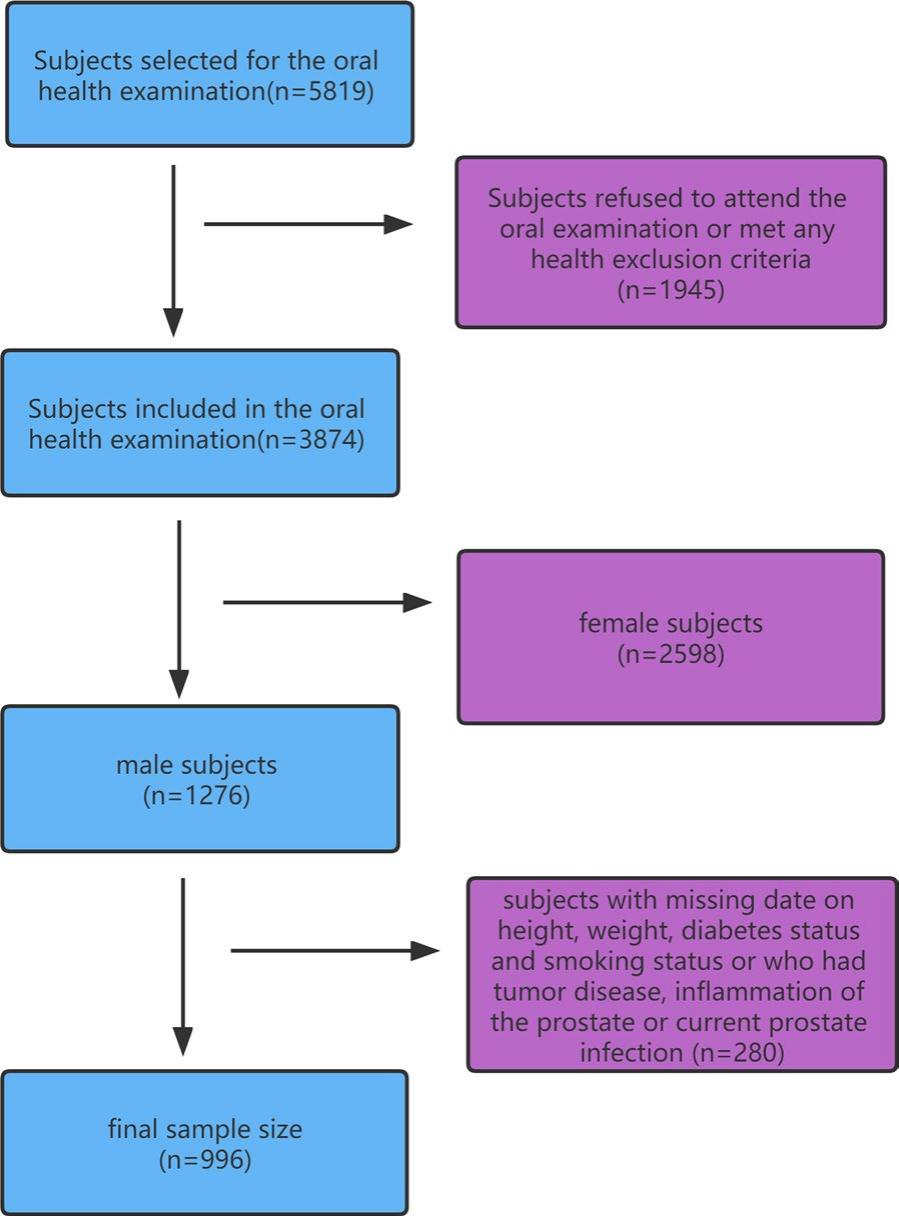
Process of sample screening for 5819 residents

After excluding 1945 residents who refused oral health examination and 2598 female subjects, we collected data about sociodemographic characteristics from questionnaires and general oral health status from oral examination for 1276 male subjects. Then, 280 men with nonnegligible missing values (height, weight, diabetes status and smoking status) or who had tumor disease, inflammation of the prostate or current prostate infection were excluded. Thus, 996 males were enrolled; the specific process of sample screening is described in detail in Fig. 1. The protocol was reviewed and approved by the Ethics Committee of Stomatology Hospital, affiliated with the Zhejiang University School of Medicine (Ethics Approval No. 2021-075). The patients provided informed consent for their data to be used for research purposes.

### Oral and periodontal examination

Oral examinations were performed by well-trained professional dentists at mobile examination centers. Before data collection, intensive examiner training was conducted to reduce measurement bias. A total of 6 dentists carried out the examinations, working in pairs, and intergroup and intragroup reliabilities were checked by calculating kappa values. Dentists were admitted to the program only if their Kappa coefficient was 0.7 or greater. Periodontal examinations were conducted according to the half-mouth, four-site protocol, randomly alternating on two diagonal quadrants (1–3 or 2–4) while excluding third molars [22]. Measurements were obtained for four interproximal sites per tooth (disto-buccal, mesio-buccal, mesio-lingual, and disto-lingual), which included probing depth (PD) (mm), gingival recession (GR)(mm), and clinical attachment loss (CAL)(mm). PD and GR were assessed using a ball-end community periodontal index (CPI) probe, and CAL was calculated as the sum of PD and GR; the result represents the distance from the cemento-enamel junction to the bottom of the pocket. Bleeding on probing (BOP) and gingival swelling were also recorded. No radiographic examination was conducted.

In epidemiological studies, periodontal status is mainly described by the use of PD and CAL assessments. Periodontitis staging was defined according to severity in none, mild-moderate, and severe periodontitis [23], using a rule-based algorithm outlined by the Centers for Disease Control and Prevention and the American Academy of Periodontology (CDC/AAP), as shown in Table 1. Importantly, these case definitions are intended for use in field surveys and not for clinical practice [24]

**Table 1:** Definitions of groups of periodontitis based on periodontitis and CAL

Periodontitis definition	Criteria
Severe	≥ 2 interproximal sites with ≥ 6 mm CAL (not on the same tooth) AND ≥ 1 or more interproximal site(s) with ≥ 5 mm PD
Mild-moderate	Among those who did not meet the severe periodontitis case definition; ≥ 2 interproximal sites with ≥ 3 mm CAL AND (≥ 2 interproximal sites with ≥ 4 mm PD (not on the same tooth) OR 1 site with ≥ 5 mm PD)
None	Meets neither the severe nor mild-moderate periodontitis definition

### Measurement of serum PSA concentration

Serum total PSA concentrations were measured as a part of routine fasting blood sample testing. Serum PSA concentrations were divided into normal and elevated levels at cutoff points of 2.5 [14] and 4.0 ng/mL, respectively. Other biomarkers, such as total cholesterol, triglycerides, and fasting plasma glucose (FPG), were also measured.

### Covariates

At the time of enrollment, questionnaires were used to collect oral health status and sociodemographic characteristics, and the subjects were divided into different categories according to different variables. There were three statuses of smoking: never (≤ 100 cigarettes), former (who quit at least 12 months ago), and current (who now smoke “every day” or “some days”). According to FPG, diabetes status was categorized as no diabetes (clinically undiagnosed diabetes; FPG < 100 mg/dL), prediabetes (clinically undiagnosed diabetes; FPG between 100 and 126 mg/dL), and diabetes (clinically diagnosed diabetes; FPG ≥ 126 mg/dL) [25]. Hypertension status was determined [26] by systolic blood pressure ≥ 140 mm Hg and/or diastolic blood pressure ≥ 90 mm Hg (average of at least three resting measurements) or by the presence of clinically diagnosed hypertension.

### Statistical analysis

Descriptive statistics were calculated to describe baseline variables classified by periodontitis severity (none, mild-moderate, and severe). We used linear regression analysis to estimate whether serum PSA concentration was associated with periodontitis severity. Two different models were used: (i) a model adjusted for age and (ii) a model adjusted for age, BMI, smoking, hypertension and diabetes. As the distribution of serum PSA concentrations was skewed, we took the natural logarithm of PSA. After adjusting for the same covariates, one ordinal regression model with elevated PSA levels was constructed for multivariate analysis. Multicollinearity was tested to eliminate individual confounding factors. The test of parallel lines was also employed to determine whether ordinal regression could be applied.

All data analyses were performed using IBM SPSS Statistics version 22.0 for Windows (IBM, Armonk, NY, USA).

## Results

The 996 participants were divided into three groups: 355 men with severe periodontitis, 479 men with mild-moderate periodontitis, and 162 men who were periodontally healthy. The characteristics are presented according to the classification of periodontitis severity in Table 2. The average age of the population was 66.88 years (standard deviation (SD) 6.09). The total mean PSA was 2.31 ng/mL, and the mean BMI was 24.59 kg/m^2^. A total of 71.1% of the participants had diabetes or prediabetes, 46.2% had hypertension, and 45.3% were former or current smokers. With respect to correlation between periodontitis and PSA, individuals with periodontitis had higher total PSA levels than those without periodontitis, regardless of severity and potential confounders.

**Table 2:** Characteristics of the participants by category of periodontitis severity (N = 996)

	Overall (N = 996)	None^a^ (N = 162, 16.3%)	Mild-moderate (N = 479, 48.1%)	Severe (N = 355, 35.6%)
Total PSA mean (SD), ng/mL	2.32 (3.69)	2.05 (2.39)	2.31 (3.39)*	2.43 (4.48)*
Age mean (SD), years	66.88 (6.09)	66.69 (6.13)	67.33 (6.12)	66.37 (6.00)
BMI mean (SD), kg/m^2^	24.59 (2.92)	24.65 (2.85)	24.66 (2.85)	24.48 (3.05)
Diabetes N (%)				
No diabetes	288 (28.9)	44 (27.2)	132 (27.6)	112 (31.5)
Pre-diabetes	438 (44.0)	73 (45.0)	217 (45.3)	148 (41.7)
Diabetes	270 (27.1)	45 (27.8)	130 (27.1)	95 (26.8)
Cigarette smoking status N (%)				
Never	545 (54.7)	108 (66.7)	260 (54.3)	177 (49.9)
Current	365 (36.6)	45 (27.8)	174 (36.3)	146 (41.1)
Former	86 (8.7)	9 (5.5)	45 (9.4)	32 (9.0)

^a^Reference category

*Statistically significant difference compared with the reference category (*p* < 0.05)

**Fig. 2:**
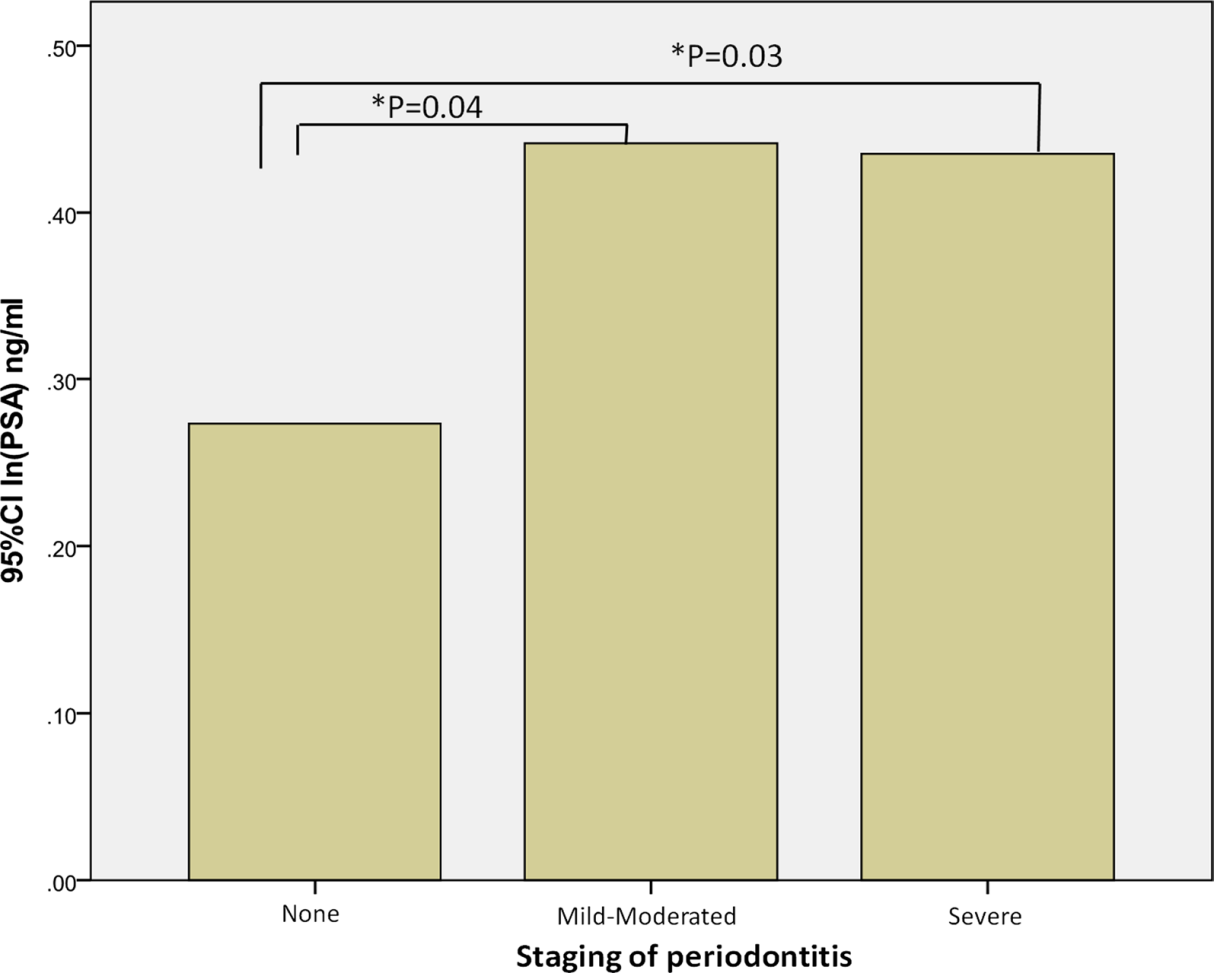
PSA levels in subjects with none/mild-moderate/severe periodontitis

After adjusting for age (Model 1), PSA levels in the blood increased with the severity of periodontitis (*P* = 0.05; Table 3). In Model 2, PSA level did not significantly correlate with periodontitis severity after adjusting for age, BMI, smoking, hypertension and diabetes (*P* = 0.06). However, PSA levels were significantly higher in individuals with mild-moderate (*P* = 0.04) and severe (*P* = 0.03) periodontitis than in those without periodontitis, as illustrated in Fig. 2.

**Table 3:** Correlation of periodontitis severity with serum PSA levels

	None (n = 162)	Mild-moderate (n = 479)	Severe (n = 355)	*P*
In(PSA) coefficient (95% CI)				
Model 1^a^	Reference	0.15 (0.01,0.30)	0.17 (0.02,0.32)	0.05*
Model 2^b^	Reference	0.15 (0.01,0.30)	0.17 (0.02,0.32)	0.06

^a^Model 1 adjusted for age in years (continuous)

^b^Model 2 adjusted for age in years (continuous), body mass index (continuous), smoking (never, former, current), diabetes (nondiabetic, prediabetic, diabetic), and hypertension (no, yes)

*Statistically significant difference when compared with the reference category

The ORs of elevated PSA did not increase with severity, regardless of whether the cutoff point was 2.5 or 4 ng/mL (Table 4). Nevertheless, individuals with PSA levels greater than 4.0 ng/mL were more likely to have periodontitis.

**Table 4:** Correlation of periodontitis severity with elevated serum PSA levels

	None (n = 162)	Mild-moderate (n = 479)	Severe (n = 355)	*P*
Odds ratio (95% CI)				
PSA > 2.5 ng/mL	39	113	91	
Model 1^a^	Reference	0.93 (0.61,1.43)	1.11 (0.72,1.74)	0.42
Model 2^b^	Reference	1.06 (0.69,1.62)	1.12 (0.72,1.73)	0.45
PSA > 4 ng/mL	19	64	51	
Model 1^a^	Reference	1.12 (0.64,1.93)	1.30 (0.73,2.30)	0.33
Model 2^b^	Reference	1.13 (0.65,1.96)	1.28 (0.72,2.27)	0.38

^a^Model 1 adjusted for age in years (continuous)

^b^Model 2 adjusted for age in years (continuous), body mass index (continuous), smoking (never, former, current), diabetes (nondiabetic, prediabetic, diabetic), and hypertension (no, yes)

## Discussion

We strove to explore the correlation between periodontitis and PSA levels in the peripheral blood in elderly Chinese men without PC to lead to a biopsy recommendation and prevent PC as early as possible. We comprehensively examined periodontal status and then explored influencing factors, including demographic characteristics, systemic disease, and lifestyle habits. After adjusting for confounding factors, PSA levels were significantly higher in men with periodontitis but did not correlate significantly with the severity of periodontitis.

Recent studies have explored the correlation between periodontitis and PSA levels. Huang et al. [27] revealed that serum PSA levels in men with periodontitis were not higher after accounting for age and other factors in a middle-aged and older population in America, and a prospective study [19] suggested no influence of chronic periodontitis treatment on tPSA or fPSA levels in asymptomatic men. However, Joshi et al. [15]*.* found that PSA levels were higher in individuals with CAL ≥ 2.7 mm and moderate/severe prostatitis than in patients with neither condition, CAL ≥ 2.7 mm or moderate/severe prostatitis. Indeed, if PSA levels increase with the severity of periodontitis or if periodontal treatment reduces PSA, we would infer a positive correlation between periodontitis and PC risk. Scholars [28] have suggested that periodontitis has a slight positive correlation with PC in a South Korean population, although another study [29] reported that in PSA-based PC screening in a US population, periodontitis did not correlate with PC risk.

Our study showed that PSA levels in individuals with periodontitis were significantly higher than those in men without periodontitis but did not correlate significantly with the severity of periodontitis. Overall, the mechanism of periodontitis and elevated PSA levels remains unclear.

Chronic inflammatory responses catalyzed by different mediators play an important role in the pathogenesis of periodontitis [30]. Studies have shown that levels of C-reactive protein [31], IL-1, IL-6 [32], and TNF-α [33] affect inflammation in the host. In the presence of inflammation, the integrity of the prostate epithelium might be compromised, causing more PSA to leak into the blood. Morote et al. [34] showed that another nonprostatic source of PSA, such as the periodontium, might increase its levels.

Recent studies [35] have reported that the risk of PC increases with age. Studies [36] based on the Chinese population indicate an incidence of PC in individuals 50–59, 60–69 and 70–79 years old of 11.6%, 16.4% and 23.1%, respectively. We chose a representative population of Chinese men with a high incidence of PSA-based PC screening, performed a dental examination and classified the severity of periodontitis rather than conducting a retrospective study, which reduced measurement error.

Several limitations of this study should be considered. First, this was a cross-sectional study that did not permit assessment of either a temporal or causal relationship between periodontitis severity and high PSA levels. Second, we excluded individuals with PC and those who reported a diagnosis of prostate disease, but we could not completely rule out the presence of undiagnosed prostate-related disease among the participants. PSA levels are elevated in patients with PC or prostatitis, suggesting a correlation between periodontitis and PSA levels. Third, a full-mouth recording includes periodontal measurements at six sites per tooth on 28 teeth, while excluding third molars is currently regarded as the gold standard for clinical examinations. Because of limited financial and time resources, our epidemiological surveys are restricted to partial-mouth recording protocols (PRP), which may result in bias [37–39]. Hence, we tried to select a method that included more information and less bias and met our resources among all PRPs. In addition, the initial age for PC screening remains controversial. Guidelines from The United States Preventive Services Task Force (USPSTF) and American Urological Association (AUA) guidelines recommend screening for men aged 55 to 70 years, whereas early diagnosis guidelines from the National Comprehensive Cancer Network (NCCN) recommend starting screening at ages 40 to 45 years and ending screening at 75 years. The European Association of Urology (EAU) guidelines recommend screening for men over the age of 50 years [40–42]. The results may have been affected by the lack of data for males 40–50 years old in our study.

## Conclusion

After adjusting for confounders, serum PSA levels in individuals with periodontitis were significantly higher than those in individuals without periodontitis in an elderly Chinese male population; however, serum PSA levels did not correlate significantly with periodontitis severity. Based on the abovementioned limitations, further studies are required to strengthen the evidence of this correlation and clarify the mechanisms whereby periodontal pathogens or the ensuing inflammation cause elevated PSA levels.

## Data Availability

After deidentification, individual participant data supporting the results reported in this article and a data dictionary are available through correspondence addressed to zfd@zju.edu.cn. Beginning at 3 months and ending at 5 years following article publication, the study protocol, statistical analysis plan and analytic code are available on request from the corresponding author for researchers who provide a methodologically sound proposal. To gain access, data requestors will need to sign a data access agreement.

## Abbreviations

PSA: Prostate-specific antigen
PC: Prostate cancer
ERSPC: European Randomized Study of Screening for Prostate Cancer
PLCO: Prostate, lung, colorectal and ovarian
CI: Confidence interval
PD: Probing depth
GR: Gingival recession
CPI: Community periodontal index
CAL: Clinical attachment loss
BOP: Bleeding on probing
CDC/AAP: Centers for Disease Control and Prevention and the American Academy of Periodontology
FPG: Fasting plasma glucose
BMI: Body mass index
OR: Odds ratio
SD: Standard deviation
USPSTF: United States Preventive Services Task Force
AUA: American Urological Association
NCCN: National Comprehensive Cancer Network
EAU: European Association of Urology
